# Association of Diabetes With Lower Back Pain: A Narrative Review

**DOI:** 10.7759/cureus.15776

**Published:** 2021-06-20

**Authors:** Shehroz Shahid, Zohaib Akhter, Mahnoor Sukaina, Fatima Sohail, Faseeha Nasir

**Affiliations:** 1 Department of Medicine, Karachi Medical and Dental College, Karachi, PAK; 2 Department of Clinical Trials Unit, University of York, York, GBR

**Keywords:** lower back pain, costochondritis, diabetes, hyperglycemia, obesity

## Abstract

Diabetes mellitus with its increasing prevalence is one of the four major non-communicable diseases. It is characterized by hyperglycemia, which may progress to chronic complications such as peripheral vascular disease and neuropathy. This paper highlights the pathophysiology associated with diabetes, which is restricted to not only hyperglycemia but also other comorbidities including chronic lower back pain.

Diabetes is a metabolic disorder associated with hypercholesteremia, hypertriglyceridemia, and hypertension. This chain of events leads to free plasma fatty acids and a pro-inflammatory state, therefore promoting calcification of blood vessels, which may block the blood supply to vertebral discs and thereby increase vulnerability in the patients with ongoing inflammatory disease such as osteoarthritis and also causing costochondritis. Functional limitation due to increased load on the weight-bearing joints is a common mechanical complication of diabetes. Obesity in diabetes is more prevalent due to a disturbed metabolism, which is aggravated with a persistent inflammatory state.

Moreover, the aim of this review is to encourage further conduction of clinical studies to explore the definite cause and potential therapy for chronic lower back pain in diabetes, thereby investigating the association of lipid metabolism and skeletal muscle atrophy leading to chronic back pain, the role of diabetic medications, and vulnerability in the female gender. Diminished physical activity and depression in diabetic patients disrupt the hypothalamic-pituitary-adrenal axis (HPA), which further contributes to lower back pain. Further clinical investigation and research in this regard will establish substantial data for the linkage between depression in diabetes and back pain. However, despite all the advancements of medical literature, the exact cause of lower back pain in diabetes is arguable.

Pain impedes the health status and life quality; therefore, it is essential to maintain the quality of health in patients with diabetes by treating not only hyperglycemia but also other multifactorial causes leading to lower back pain.

## Introduction and background

According to the World Journal of Diabetes, diabetes mellitus is a chronic metabolic disorder in which the body's ability to produce or reciprocate to insulin is lost, resulting in impaired carbohydrate metabolism and increased levels of glucose in the blood [1]. Diabetes is among the four chief non-communicable diseases (cardiovascular disease, diabetes, cancer, and chronic respiratory disease) with a growing burden of 382 million people living with type 2 diabetes and an increasing prevalence worldwide [2]. As highlighted by the International Diabetes Federation, the global prevalence of diabetes was estimated to be 463 million in the year 2019, rising to 578 million by the year 2030 and around 700 million by 2045 [3]. China, India, and the United States have the largest population of diabetes, with 116, 77, and 31 million people, respectively [3]. In adults, the highest prevalence of diabetes is in the Middle East and North Africa (10.9%), though the highest number of adults diagnosed with diabetes was in the Western Pacific region (37.5%) [1]. The age attunement death rate associated with diabetes increased to 165.83 per 100,000 in 2016 from 150.43 per 100,000 in 2016, with women having a higher mortality rate as compared to men [4].

Diabetes is mainly of two types; type 1 diabetes mellitus, also called insulin-dependent which occurs due to the destruction of insulin-secreting beta cells and lack of insulin secretion most common in children and adolescents, and type 2 diabetes mellitus, which comprises 90% of the diabetic cases, is non-insulin-dependent, caused by impaired sensitivity of target tissue receptors to insulin, and is most commonly associated with obesity [5].

In diabetes, long-standing hyperglycemia leads to both microvascular and macrovascular chronic complications. Nephropathy, peripheral neuropathy, and retinopathy are microvascular complications, and peripheral vascular disease, cerebrovascular disease, and ischemic heart disease are macrovascular complications [6]. Both type 1 and type 2 diabetes lead to neuropathy in around 90% of the patients within 25 years of their diagnosis with signs and symptoms that include muscle weakness, lack of coordination, and pain gradually spreading from the lower side of the body to the back and neck [7,8]. Diabetes and low back pain are somehow connected, and pain is one of the leading symptoms of diabetic neuropathy [9].

In the medical literature, many possible causes are associated with the incidence of lower back pain in diabetes including costochondritis, pain-sensitive structures around the lumbar vertebrae, obesity, decreased muscle strength, depression, and an inactive lifestyle [10-12].

## Review

Increased risk of cartilage inflammation

In diabetics, osteoarthritic cartilages are more reactive to pro-inflammatory stress as compared to non-diabetics [11]. Diabetic patients manifest inflammatory phenotype possibly due to damage to the beta cells and target receptors of insulin caused by chronic hyperglycemia and Interleukin-1 beta (IL-1β) stress. Besides this, other factors might also involve increased responsiveness of cartilages to inflammatory stress such as type 2 diabetes which is characterized by hyperglycemia along with hyperinsulinemia, insulin resistance, and an increase in free fatty acids [13]. These aspects are responsible for costochondritis including increased expression of glucose transporter (GLUT), increased glucose uptake, oxidative stress, and polyol pathway, which is activated during hyperglycemia.

Hyperglycemia and hypercholesterolemia in type 2 diabetes are the major contributors to the cartilaginous and calcifying lesions of blood vessels [14,15]. Since intervertebral discs are avascular, disc cells depend on the disc's marginal blood vessels for supply of nutrients and removal of metabolic waste products such as lactic acid [16]. Impaired blood flow, as followed by vascular calcification, can also be the source of an increased likelihood of costochondritis.

Cartilage inflammation, tissue remodeling, matrix destruction and stiffness, and chondrocyte destruction are mediated by advanced glycation end products (AGEs) [17]. AGEs are the detrimental compounds formed by the combination of fat or protein with sugar in the bloodstream. Synthesis of the matrix, energy production, and homeostasis in cartilages is maintained by glucose [18]. AGEs formation is notably increased in degenerative diseases, mainly in diabetes, atherosclerosis, chronic kidney disease, and Alzheimer's disease [19]. Peroxisome proliferator-activated receptor gamma (PPARγ) is a nuclear receptor type 2 and plays an important role in sensitization of insulin and peripheral glucose metabolism [20,21]. PPARγ is known to play a vital part in the pathogenesis of diabetes and inflammation [22]. In addition, PPARγ is an anti-inflammatory agent that decreases cartilage degradation by inhibiting cytokines and chemo-attractants [19,23]. Hence, the role of PPARγ in cartilage damage due to hyperglycemia is not clear [24].

Obesity: a contributing factor?

Diabetes is significantly linked with the accumulation of excess fat causing obesity, which causes various adverse effects on the health status of the patients [25]. Obesity causes an increase in mechanical stress and a pro-inflammatory; therefore, the association between obesity and chronic joint pain is very well established [26-28].

The biochemical changes due to the unusual distribution of adipose tissue in the body particularly the trunk can cause a series of spine disorders. The increased mechanical load on the lumbar spine can develop diseases such as intervertebral disc degeneration, disc herniation, and spinal stenosis with the shared symptom of lower back pain [26]. In elder obese people, the likelihood of chronic back pain becomes twice as compared to young adults [28]. Usually, people reporting musculoskeletal pain tend to have increased fat mass and decreased lean mass as compared to those not reporting the pain [29]. A community-based survey reported that various pain types, including lower back pain and chronic widespread pain, are widely associated with obesity [30].

There are existing guidelines for weight loss and improvement in diabetes outcomes, but there are not enough genetic data to support decisions for the management of obesity associated with type 2 diabetes [31].

Loss of muscle strength

Type 2 diabetes in higher age groups shows an increased reduction in lean mass of legs and decreased muscle strength and functional competency as compared to the people having a normal amount of glucose in the blood [32]. In type 2 diabetes with or without neuropathy, maximal muscle strength is deteriorated and progressive physical disabilities with impaired mobility are seen [33]. Disturbed lipid storage is an important contributing factor in the development of muscle atrophy along with insulin resistance [34]. It may be due to the accumulation of harmful lipid derivatives in skeletal muscles and irregular mitochondrial function particularly in type 1 muscle fibers, which are dependent on the metabolic action of insulin [35]. Unfortunately, the relationship between lipid accumulation in type 2 diabetes and muscle atrophy remains largely unexplored.

Increased activity of the polyol pathway, accumulation of AGEs, production of reactive oxygen species, and impaired vascular function are important factors for diabetic polyneuropathy, which can result in reduced muscle strength due to muscle atrophy [36]. Decreased muscle strength in type 2 diabetes may also be associated with a diminished quality of life [37].

Loss of muscle strength, particularly in the legs, in patients with type 2 diabetes could be one of the important contributing factors in developing chronic lower back pain, as lack of support leads to constant wear and tear due to extra load on the intervertebral disc [38]. Therefore, musculoskeletal pain management is of great significance in the lifestyle intervention of type 2 diabetes [39].

Depression and lack of physical activity

Diabetes along with depression is a devitalizing condition associated with significant morbidity and mortality [40]. The coexistence of diabetes with depression is linked with poor adherence to therapy, loss of metabolic control, decreased standard of living, and, most importantly, increased disability, lack of physical activity, and productivity. The 60% increased risk of depression is associated with type 2 diabetes [41]. Lately, it was believed that depression in diabetes is a result of diabetic complications. According to recent studies, only diagnosed diabetic patients show an increased rate of depression suggesting the fact that depression in diabetes is due to the burden of managing its complications and not because of hyperglycemia [12].

Physiologically, hypothalamic-pituitary-adrenal axis (HPA axis) dysfunction is an important link between stress, depression, and diabetes [42]. Dysregulated HPA axis results in hypercortisolism, disturbed diurnal rhythm, and increased inflammation, which possibly could be the cause of lower back pain [12]. Correspondence between depression and increased inflammation by HPA axis dysfunction makes depression a cofounder for back pain in diabetes. Individuals with both depression and diabetes have a functional disability, resulting in poor posture causing back pain and lack of substantial activities more, as compared with the individuals struggling with diabetes and depression alone [43-45]. However, more studies are required to establish the exact relationship between them.

Role of diabetic medications

Diabetic medications such as insulin may affect musculoskeletal function and blood flow [46]. These medications possibly influence pain due to their glycemic control effects and analgesic properties, as the impact of insulin in losing muscle mass is very well known [47]. A study has reported that treatment of diabetes with dipeptidyl peptidase-4 (DPP-4) inhibitors may increase pain in the joints [48]. The role of these diabetic medications in causing lower back pain should be investigated further.

## Conclusions

Diabetes and lower back pain both can have a considerable impact on a patient's life when acting alone and or in combination, causing severe health deterioration. Comprehensively, the relation of diabetes with lower back pain and disc degeneration remains a controversial subject in the literature of medicine. Evidence supports that back pain in diabetes is multifactorial rather than just being caused by one factor alone. This review aimed to highlight the need to understand the exact cause of lower back pain in diabetes. Individuals with lower back pain have an unhealthy lifestyle, therefore leading to a deteriorated health status. As the intensity of pain becomes severe in these patients, pain medications are required often. Therefore, it is important to explore more about the factors associated with lower back pain in diabetes, which would enable the health care providers to design preventive strategies for such patients. Until now, dietician consultation, physical activity, weight management, and exercise are considered good practices for diabetic patients.

A study confirmed that the risk factors for having lower back are different in men and women. However, sex-based differences in lower back pain further need to be investigated.

We suggest that the healthcare providers should look into the effect of diabetic medications as it is well supported by the literature that these medications affect the intensity of pain and therefore the in-depth connection should be explored.
